# Can Brief Empathy Training Increase Sexual Harassment Bystander Intervention Intentions?

**DOI:** 10.3390/bs16020227

**Published:** 2026-02-04

**Authors:** Tristan Barta, Zachary E. Piper, Harshit Chaubey, Jessica Kiebler, Margaret S. Stockdale

**Affiliations:** Department of Psychology, Indiana University Indianapolis, 402 N. Blackford St., Indianapolis, IN 46202, USA; tbarta@iu.edu (T.B.); zepiper@iu.edu (Z.E.P.); hchaubey@iu.edu (H.C.); jkiebler@iu.edu (J.K.)

**Keywords:** sexual harassment, empathy, perspective taking, training, bystander intervention, gender

## Abstract

Sexual harassment (SH) remains widespread in workplaces and academic settings. Traditional compliance-based training has shown limited success in preventing SH or motivating bystander intervention. This study developed and tested a brief online empathy training module that can be completed in under 30 min that aims to help participants better understand and support people who experience SH and increase their willingness to intervene. Two experiments were conducted with U.S. adults recruited from the CloudResearch Connect platform (Study 1: 122 men and 140 women; Study 2: 132 men, 112 women, 4, other gender) who were randomly assigned to complete the SH empathy module, another empathy training module (burglary empathy training), time management training, a standard SH training module, or a waitlist control condition. Measures assessed empathy, perspective taking, and bystander intervention intentions. In Study 1, empathy correlated with bystander intentions, but there were no significant group differences; women reported higher empathy and bystander intentions than men. In Study 2, participants who received SH empathy training demonstrated higher empathy and perspective taking than those in other groups, and empathy improvements were associated with greater willingness to intervene. Gender did not moderate these effects. Overall, findings suggest that integrating a short empathy module into SH prevention programs can enhance readiness to act as supportive bystanders. Future research should assess the longevity of these effects and whether they translate into real-world behavioral change.

## 1. Introduction

Sexual harassment (SH), defined as “unwelcome sexual advances, requests for sexual favors, and other verbal or physical conduct of a sexual nature … that is so frequent or severe that it creates a hostile or offensive work environment or when it results in an adverse employment decision affects an individual’s employment, unreasonably interferes with an individual’s work performance, or creates an intimidating, hostile, or offensive work environment” (E.E.O.C., 1997), remains a persistent challenge in workplaces, particularly in male-dominated industries and academic or other training environments. Large-scale surveys indicate that between one-quarter and one-half of women experience SH in their careers, with the highest rates occurring in the military, academia, medicine, and law enforcement (Fitzgerald et al., 1999; Henning et al., 2017; Lonsway et al., 2013; U.S. Government Accountability Office, 2020). Although men report lower prevalence rates, individuals who identify as LGBTQ+ or who belong to racial or ethnic minority groups often face heightened risk (Clancy et al., 2014; U.S. Government Accountability Office, 2020). Harassment is especially prevalent in contexts where power imbalances and unclear reporting structures create conditions of vulnerability, such as graduate training programs and field research sites (Ang & Schneider, 2021; Clancy et al., 2014). Despite growing public awareness, evidence shows that the incidence of SH has not significantly declined over time (Feldblum & Lipnic, 2016), underscoring the limits of existing prevention efforts.

To address this, organizations have relied heavily on training programs aimed at reducing misconduct. Yet, traditional approaches that focus narrowly on legal compliance and definitions of harassment have produced limited and inconsistent changes in attitudes, behavioral intentions, and endorsement of harassment myths (Roehling et al., 2022). We refer to such training programs collectively as “standard practice” (standard) or “compliance-based” SH training. Meta-analytic evidence suggests that conventional training tends to be most effective among women and those with egalitarian beliefs, whereas men often remain resistant unless training appeals to shared values or empathy (Dobbin & Kalev, 2019; Perry et al., 2019). In response, researchers have highlighted the promise of empathy-based approaches that cultivate emotional understanding and perspective-taking toward SH targets. By fostering empathic concern, these programs can reduce victim-blaming and trivialization, strengthen moral motivation, and enhance bystander intervention intentions (Blahopoulou et al., 2025; S. Y. Lee et al., 2019; Liang & Park, 2022). These developments have paved the way for innovative training programs that integrate empathy as a central mechanism to improve both attitudinal and behavioral outcomes in SH prevention.

Empathy training is not new. Dating back to the 1970s, behavioral scientists have created and tested empathy-based interventions focused on changing the hearts and minds of instigators of interpersonal harm including sexual assault, sexual harassment, and adolescent aggression, among other purposes (e.g., Foubert & Newberry, 2006; Kipper & Ben-Ely, 1979; Marshall et al., 1996; Pecukonis, 1990). However, the complexity and duration of empathy training programs vary widely. Traditional programs often span multiple sessions with structured practice (e.g., 12 h over three weeks), while others condense training into multiple short modules of 30 min or less (Janoka & Scheckenbach, 1978; Pecukonis, 1990). Importantly, though, meta-analytic evidence indicates no clear relationship between program length and effectiveness; both brief and extended interventions can yield moderate gains in empathy (Teding van Berkhout & Malouff, 2016).

More recently, immersive media, including 360° video and virtual reality (VR) using head-mounted displays (HMDs; VR headsets worn like goggles), have been tested as novel empathy-enhancing tools. Studies suggest VR can increase state empathy and related prosocial variables, though findings vary by device and design (Herrera et al., 2018; Y. Lee et al., 2024; Martingano et al., 2021; Martingano et al., 2023). These approaches leverage presence (feeling physically present in the same environment as the subject) and embodiment (virtually inhabiting the body of another person)—concepts widely used in virtual reality contexts (Ventura et al., 2021)—to induce perspective-taking in ways that are not possible with traditional didactic content. However, technological complexity alone does not guarantee superior outcomes. Reviews show inconsistent benefits of HMD VR compared to 2D or 360° video when narrative content and perspective-taking tasks are held constant (Martingano et al., 2021; Martingano et al., 2023; Trevena et al., 2024). Indeed, several studies suggest narrative quality, framing (e.g., focusing on victims, alleged perpetrators, or others), and structured reflection exercises may influence impact more significantly than the level of immersion (Shin, 2018; Young et al., 2022).

The purpose of our research was to develop a brief (~30 min), online-capable, empathy training module for sexual harassment training and to test it for its efficacy in enhancing trainees’ behavioral intentions to act proactively on behalf of others who have experienced sexual harassment—a concept known as bystander intervention (Liang & Park, 2022). Consistent with the Theory of Planned Behavior (Ajzen, 1991), verbal intentions to behave are a critical determinant of actual behavior. The training module capitalized on the importance of personally relevant narratives of sexual harassment followed by structured reflection exercises. Across two studies we tested the effectiveness of this training in comparison to empathy training in a non-harassment context (victims of burglary), non-empathy training in a non-harassment context (time-management training), standard compliance-based SH training, and a non-training, waitlist control condition on bystander intervention intentions. We start with a brief review of empathy and its close cousin, perspective taking, and their importance for compelling behavioral change; then, we present our hypotheses.

### 1.1. Empathy and Perspective Taking

Empathy and perspective taking are distinct yet interrelated socio-cognitive constructs central to understanding human social functioning. Empathy is broadly defined as an affective response that stems from the apprehension or comprehension of another’s emotional state and is congruent with that state (Decety & Jackson, 2004; Eisenberg & Eggum, 2009). Subcomponents of empathy include empathic concern—an other-oriented emotion of care and compassion for others in distress—and personal distress (or empathic distress)—a self-oriented response of discomfort or anxiety upon witnessing another’s suffering (Eisenberg & Eggum, 2009; Niezink et al., 2012). Sympathy shares similarities with empathic concern but is often described as an affective reaction involving sorrow or concern for another’s suffering without necessarily experiencing their emotions (Niezink et al., 2012). In contrast, perspective taking is the cognitive capacity to adopt another’s point of view, a process facilitated by executive functions and supported by neural networks including the medial prefrontal cortex and temporoparietal junction (Decety & Jackson, 2004).

Empirical research consistently supports the role of both empathy and perspective-taking in reducing harmful behaviors and increasing prosocial responses. Perspective-taking has been shown to decrease stereotyping and prejudice (Galinsky et al., 2008; Todd & Galinsky, 2014), improve intergroup attitudes (Stephan & Finlay, 1999), and enhance interpersonal sensitivity (Batson et al., 1997). In the context of sexual harassment and assault, fostering empathy for victims shifts attitudes and reduces victim-blaming (Bongiorno et al., 2020). Training programs that emphasize perspective taking have been found to increase the likelihood of bystander intervention and reduce acceptance of rape myths and sexually aggressive behaviors (Pithers, 1999; Shih et al., 2009). Fabi et al. (2019) further demonstrated that empathic concern predicted moral sensitivity and prosocial behavioral intentions in organizational contexts. Across this body of work, a consistent pattern emerges. When individuals engage in affective empathy and cognitive perspective taking, they are more likely to inhibit harmful actions and instead act in ways that promote social justice.

Empathy training programs employ a range of instructional elements that vary in effectiveness depending on how well they activate cognitive, affective, and behavioral aspects of empathic functioning. Perspective-taking exercises, such as imagining the experiences of others or engaging in role play, consistently emerge as effective tools for promoting cognitive empathy and reducing harmful behaviors such as prejudice and sexual aggression (Batson et al., 1997; Todd & Galinsky, 2014). Training approaches that evoke emotional engagement—through video narratives, simulations, or VR—appear particularly effective in eliciting empathic concern and fostering prosocial action, including bystander intervention (Foubert & Newberry, 2006; Trevena et al., 2024). Programs grounded in experiential learning (e.g., psychodrama, Carkhuff’s humanistic model) that combine emotional alignment, feedback, and perspective-taking tend to show stronger and more sustained outcomes than didactic instruction alone (Janoka & Scheckenbach, 1978; Kipper & Ben-Ely, 1979). Meta-analytic evidence supports the superior efficacy of multi-component interventions, particularly those that integrate reflection, emotional activation, and interpersonal skills practice, in building empathy and promoting behavioral change (Teding van Berkhout & Malouff, 2016). Our primary hypothesis is that compared to control conditions, the sexual harassment empathy training module will be positively associated with measures of empathy and perspective taking which, in turn, will be positively associated with self-reported intentions to engage in bystander intervention in future instances of sexual harassment.

### 1.2. Gender Differences

There is evidence that there may be self-identified gender differences in the expression of empathy and in the effectiveness of empathy training for motivating prosocial behaviors, such as bystander intervention. Research consistently shows small-to-moderate gender differences in empathy, with women generally reporting higher levels of empathic concern, personal distress, and emotional responsiveness, as well as modest advantages in emotion recognition, while cognitive empathy or perspective-taking differences are smaller and less consistent (Christov-Moore et al., 2014; Mestre et al., 2009; Pang et al., 2023; Thompson & Voyer, 2014). These baseline distinctions shape responsiveness to empathy training in the context of sexual harassment, sexual assault, and related forms of aggression. For women, empathy exercises often increase negative affect and reduce rape-myth acceptance, which in turn predicts greater bystander intervention behaviors (Hines et al., 2019). For men, tailored interventions that emphasize survivor narratives and concrete victim harm, frame intervention as a moral or group-based responsibility and incorporate active perspective-taking or planning are effective in reducing harassment myths and self-reported likelihood to offend, while also increasing bystander intentions (Diehl et al., 2014; Foubert & Newberry, 2006; Schewe & O’Donohue, 1993). Importantly, both brief inductions and more immersive methods (e.g., 360° video, VR) can enhance empathy and bystander outcomes, but meta-analyses and experimental comparisons suggest that the narrative content, framing, and reflection components matter more than technological complexity or program length (Martingano et al., 2021, 2023; Rawski et al., 2022; Ventura et al., 2021). Taken together, these findings underscore that while women’s affective empathy, compared to men’s, may lead to prosocial responding, carefully designed, concise, and contextually framed empathy training can effectively engage men and promote meaningful changes in prosocial attitudes and behaviors toward sexual harassment and other forms of aggression.

Gender differences in bystander intervention intentions and related behaviors are more nuanced. Systematic reviews indicate that women report a greater willingness to help than men across a range of sexual-violence scenarios, but the effects are mixed (Mainwaring et al., 2023). Some studies with college students show that men reported higher bystander intentions than women (Amar et al., 2014). Kettrey and Marx’s (2019) meta-analysis of bystander programs found little evidence that gender moderated the effects of training effectiveness on bystander intentions and bystander efficacy. We included participant gender as a study variable but made no specific hypotheses.

### 1.3. Purpose of the Current Research and Hypotheses

We sought to develop a empathy training module that could be added to existing sexual harassment training programs that would (a) feature key elements of effective empathy training programs, including the use of personally relevant narratives of sexual harassment victims, empathy-induction exercises, perspective-taking exercises, and personal reflection; (b) be scalable for either in-person or online training modalities; and (c) be relatively brief so that it could be added to existing training programs. Our intention was not to replace standard, compliance-based sexual harassment training programs that address other important goals such as defining sexual harassment, explaining policy elements, instructing trainees how to avoid harassing conduct and how to report such conduct (Roehling et al., 2022). Rather, our goal was to enhance standard sexual harassment training with empathy training to improve behavioral outcomes such as bystander intervention intentions.

We tested the following hypotheses:

- Hypothesis 1: Self-report ratings of empathy and perspective taking for sexual harassment victims will be higher for participants who engage in sexual harassment empathy training compared to participants who engage in empathy training for a non-relevant context (burglary empathy training) or who engage in non-empathy training (time management training).
- Hypothesis 2: Empathy and perspective taking will be positively correlated with bystander intervention intentions.
- Hypothesis 3: Empathy and perspective taking will mediate the effect of empathy training conditions on bystander intervention intentions.
- Research Question 1: Will there be gender differences in self-reported empathy, perspective taking, and bystander intervention intentions?
- Research Question 2: Will gender moderate the effectiveness of empathy training conditions on empathy, perspective taking, and bystander intervention intentions?

## 2. Study 1

In Study 1, we developed an empathy training module for sexual harassment and compared it to a similarly structured training program for a non-sexualized and non-gendered yet distressing context–burglary. These conditions allow us to test whether empathy training of any sort produces empathy toward sexual harassment victims. We also compared our empathy training to a non-empathy, non-harassment training context—time management training—on increasing intentions to intervene in an SH context.

### 2.1. Study 1 Methods

#### 2.1.1. Participants

We recruited 270 adults residing in the U.S. through the CloudResearch Connect platform, which has demonstrated reliability for online research (Douglas et al., 2023). Eight were excluded due to zero variance on responses to the mediator or dependent variable items. Of the participants in the final data (*n* = 262), 53% (*n* = 139) defined their gender as “woman” and 47% (*n* = 123) defined their gender as “man.” Most (69%, *n* = 181) of the participants identified as White, 15% (*n* = 39) of the participants identified as Black, 10% (*n* = 26) of the participants identified as Asian/Asian American, 7% (*n* = 18) identified as Hispanic/Latina/o, and 3% (*n* = 8) identified with two or more races. The average age of the participants was 41.6 yrs (*SD* = 13.17). Participants had varied educational backgrounds: 43% (*n* = 113) held a bachelor’s degree, 15% (*n* = 39) a master’s degree, 11% (*n* = 29) a high school diploma or GED, 10% (*n* = 26) an associate’s degree, and 5% (*n* = 13) a professional or doctorate degree. Most (63%, *n* = 163) were employed full time for pay, 14% (*n* = 37) were employed part time, 13% (*n* = 34) were unemployed, and the remaining 11% (*n* = 29) identified as a “gig worker,” seasonal employee, or other. Of those who registered occupational and relationship status with CloudResearch (voluntary information for registered Connect platform participants, *n* = 270^1^), 9% (*n* = 23) were employed in information technology, 8% (*n* = 21) in education and training, 7% (*n* = 19) in medicine, 6% (*n* = 17) in science, technology, engineering & math (STEM), 6% (*n* = 16) in retail, and less than 6% each in other occupations, or were retired (16%, *n* = 43 listed “other”). Regarding relationship status, 37% (*n* = 99) of participants were married, 31% (*n* = 84) were single, 16% (*n* = 42) said they were in a relationship, and 7% (*n* = 23) were divorced (less than 3% each indicated some other status).

#### 2.1.2. Measures

Empathy was measured with a 15-item scale adapted from Batson et al. (1997), in which participants rated on a 7-point scale, ranging from 1 (not at all) to 7 (extremely), the extent to which each adjective describes their feelings toward SH victims. The scale measures two forms of empathy. Empathic concern was measured with eight adjectives, such as sympathetic, softhearted, and warm. *α* = 0.88. Personal distress was measured with seven items such as grieved, perturbed, and irritated. *α* = 0.83.

Perspective Taking for Sexual Harassment (PTSH) was measured with a 6-item scale developed by Ventura et al. (2021), in which respondents answered the extent to which they agreed with statements about taking the perspective of SH victims. Sample items were “Do you ever imagine how you would act if you were a victim of SH?” and “To what extent do you understand the emotions of SH victims while they are being harassed?” The items were rated by respondents from 1 (not at all) to 5 (totally) on the extent they experienced each of the situations or agreed with the statement. *α* = 0.87. After collecting data, we realized that we made a programming error such that only participants in the SH empathy condition completed this scale. Therefore, we were unable to use this measure in our analyses.

Sense of oneness with SH victims was measured with Aron et al.’s (1992) Inclusion of Other in the Self Scale which displayed seven increasingly overlapping circles where one circle was labeled “You” and the other was labeled “SH victims.” The circles varied in overlap, and respondents were asked to select one of the pictures that corresponded to one of the items that closely symbolizes their sense of oneness with the victim. The participants’ answer choices ranged from image 1 to image 7. Image 1 represented no sense of oneness with the victim, which was shown in the picture as having no overlap, and image 7 represented almost complete oneness with the victim.

Bystander intervention intentions were measured using a 10-item scale adapted from Liang and Park (2022), which operationalizes intentions as a rating of the likelihood of engaging in different behaviors if they had witnessed a coworker or student experiencing SH. Items included responses such as “report the incident to someone in a higher position” or “ask the initiator (perpetrator) to refrain from such behavior.” One item, “pretend that you did not witness the incident,” was not included in the scale score because it does not reflect a positive bystander intervention intention. Responses were recorded on a 5-point scale ranging from 1 (not at all likely) to 5 (extremely likely)*.* High scores indicated intentions to intervene. *α* = 0.88.

#### 2.1.3. Procedures

The study design was an online randomized, post-test only experiment. We did not include pre-tests so as not to sensitize our participants to the measures before training. The study was carried out via a Qualtrics survey through CloudResearch’s Connect online platform. After the participants had read the study information sheet and had consented to participate in the study, they watched a short video introducing them to the study. Next, they were randomly assigned to one of the three training conditions: time-management (*n* = 88, 56.8% women, *M_age_* = 38.8 yrs), burglary empathy training (*n* = 85, 44.7% women, *M_age_* = 40.5 yrs), and sexual-harassment empathy training (*n* = 89, 58.4% women, *M_age_* = 42.7 yrs).

The SH empathy training began with a prompt to think about someone other than themselves who had experienced SH. This may be someone they knew or someone they heard about, e.g., in the media. They were asked to describe what happened, who did it, where it occurred, and how it affected them. We did not define SH for them; instead, we allowed them to use their own definition. They answered a yes/no question about whether they were able to think about a real story of sexual harassment. All participants in this condition answered “yes.” Next, they were prompted to think about the incident again, but to retell it as if it happened to them. The prompt read: “Now, think about this same incident, but imagine that YOU are the person who experienced this sexual harassment. Describe the harassment in first-person and present tense, as the person experiencing sexual harassment that you described in your answer to the question above in present tense, e.g., “I am attending a meeting for work, and ‘Joe’ comes up to me and says …”.

For the next prompt, participants were to continue taking the perspective of the person experiencing the harassment and to identify as many short-term consequences as possible that resulted. The prompt read: “Continuing to take the perspective of a person experiencing sexual harassment, in the first person, think about everything that happened to you and how those experiences affected you immediately following the incident. Identify as many of the short-term consequences that resulted from those events, using 10 or more words. These consequences may be emotional, psychological, professional/academic, social, etc. For example, you might say, shortly after the harassment, I was … (I felt …, I experienced …).” The next prompt had them identify long-term consequences, still taking the victim’s perspective with the prompt: “Now, consider the more lasting ramifications brought about by those experiences. Identify as many of the long-term consequences that resulted from those events, using 10 or more words. These consequences may be emotional, psychological, professional/academic, social, etc. For example, you might say, several months (years?) after the harassment I felt …, I experienced …, etc.).”

After completing the empathy exercises, participants were asked to reflect on their own experiences during the training with the following prompts: “What emotions and thoughts were evoked when you wrote about someone else’s sexual harassment experiences? In other words, how did it feel to recount the experience and the aftermath as if it happened to you?” “What did you learn from this story and exercise with sexual harassment?” “What new insights regarding sexual harassment did you get from this story?”

The burglary empathy training followed the same format except that respondents were asked to recall an incident of burglary and to engage in the empathy exercises and reflection exercises in the context of being a burglary victim. The purpose of the burglary condition was to compare empathy training for two distressing events (SH vs. burglary) to assess whether training for empathy for a non-gendered or non-sexualized experience would be as effective as empathy training for sexual harassment. The time management training, which was used as a non-empathy training condition, had participants write three sentences about habits that waste time, followed by a brief description of SMART goals (specific, measurable, achievable, relevant, and time-bound). They were prompted to state a goal to improve their time management using the SMART goal format. They then identified strategies aligned with that SMART goal by breaking tasks or sub-goals into categories: urgent and important, important but less urgent, urgent but not important, and neither urgent nor important. Finally, they were prompted to identify ways to hold themselves accountable, such as sharing the goals with others, and rewarding themselves for reaching each of their sub goals and the final goals.

At the end of their respective training session, participants completed the empathy measures and oneness scale (presented in random order), and bystander intervention intentions scale. Participants then completed a standard set of demographic questions and then received the debriefing statement at the end. Participants in all three conditions were paid $2.50 for their participation. The study was approved by our Institutional Review Board as an exempt protocol.

### 2.2. Study 1 Results

To verify that our SH empathy training was brief, we calculated the average time that participants spent on the study (we did not time the actual training, therefore our time estimates are liberal). Eliminating outliers who were 2 *SD* above the mean, the average duration for those in this condition to complete the study was 16.97 min (*SD* = 6.67 min).

Table 1 presents the means, standard deviations, one-way ANOVA results, and intercorrelations among the study variables. Bystander intentions were significantly positively correlated with all three measures of empathy—empathic concern, empathic distress, and oneness—supporting hypothesis 2. However, there were no significant differences among these variables between the three training conditions, although the means were highest in the SH empathy condition, failing to support hypothesis 1.

For Study 1, formal mediation tests were not conducted. Although empathy measures correlated with bystander intentions, no significant between-condition differences on empathy or intentions were observed, and the PTSH measure was administered only to participants in the SH-empathy condition due to a programming error. Because prerequisites for mediation were unmet, we did not proceed with analyses with PROCESS (Hayes, 2022) for Study 1.

There were significant gender differences on bystander intervention, *t* = 3.20, *p* = 0.002, Cohen’s *d* = 0.40, with women reporting greater intentions to intervene (*M* = 4.21, *SD* = 0.60) than men (*M* = 3.95, *SD* = 0.69); empathic concern, *t* = 2.17, *p* = 0.030, Cohen’s *d* = 0.27, with women expressing greater empathic concern (*M* = 5.62, *SD* = 0.95) than men (*M* = 5.36, *SD* = 1.05); and oneness, *t* = 4.61, *p* < 0.001, Cohen’s *d* = 0.57, with women expressing higher oneness with SH victims (*M* = 4.26, *SD* = 1.71) than men (*M* = 3.31, *SD* = 1.58). These findings affirmatively answer research question 1 regarding gender differences in empathy and bystander intentions. However, gender did not moderate the (lack of) effects of the training conditions on these variables, answering research question 2 in the negative.

### 2.3. Study 1 Discussion

The goal of Study 1 was to conduct an initial test of a brief, scalable empathy-based sexual harassment (SH) training module and to examine whether it enhanced empathy-related responses and bystander intervention intentions relative to comparison conditions. Although the hypothesized between-condition differences were not supported, Study 1 yields several theoretically and practically informative findings that guide the design of Study 2.

Consistent with prior research cited below, empathic concern, empathic distress, and a sense of oneness with SH victims were each positively associated with bystander intentions, although the magnitudes of the correlations were modest. This pattern aligns with the substantial literature, demonstrating that empathy—particularly empathic concern and perspective taking—motivates prosocial behavior by increasing moral sensitivity, reducing victim blame, and heightening responsibility for action, including bystander intervention in harassment and sexual violence contexts (Batson et al., 1997; Fabi et al., 2019; Kettrey & Marx, 2019). These findings reinforce empathy as a psychologically meaningful mechanism through which individuals become motivated to act on behalf of others who experience harm.

Despite these associations, the SH empathy training did not produce significantly higher empathy or bystander intentions than the burglary empathy or time-management conditions. Inspection of the descriptive statistics suggests that empathic concern and bystander intervention intentions were near the upper bounds of their respective scales across all conditions, whereas empathic distress showed greater dispersion and less evidence of ceiling effects. This pattern may have constrained the ability to detect between-condition differences for empathic concern and bystander intentions, while also suggesting that brief interventions may differentially influence components of empathy. We also speculate that ratings on our prosocial measures of empathy and bystander intentions may have been influenced by motivations to respond in socially desirable ways, which we attempted to address in Study 2.

Study 1 also revealed reliable gender differences, with women reporting higher empathic concern, greater oneness with SH victims, and stronger bystander intervention intentions than men. These findings replicate a robust body of research showing that women, on average, report higher affective empathy and greater willingness to intervene in sexual harassment and sexual violence scenarios (Christov-Moore et al., 2014; Hines et al., 2019; Mainwaring et al., 2023; Thompson & Voyer, 2014). Importantly, gender did not moderate the effects of training condition, suggesting that the absence of condition main effects was not due to men’s and women’s opposing responses to empathy or bystander intervention intentions.

Taken together, Study 1 suggests that empathy is linked to bystander intervention intentions but that detecting training effects requires more precise comparisons and measurement. Study 2 was therefore designed to strengthen the experimental test by replacing the burglary empathy and time-management conditions with two controls commonly used in training evaluations: a standard, compliance-based SH training program, which serves as a “standard practice” control condition, and a waitlist control condition. Study 2 also corrects the programming error by assessing SH-specific perspective taking across all conditions, adds a multidimensional empathy measure capturing affective empathy, cognitive empathy, and sympathy, and includes a measure of socially desirable responding to evaluate whether reported empathy and bystander intentions are influenced by impression management concerns. These design enhancements allow for a more rigorous test of whether empathy-based SH training uniquely enhances the psychological processes that motivate bystander intervention. Study 2 tested each of our hypotheses and research questions. Hypothesis 1 was modified as follows:

- Hypothesis 1 (modified): Self-report ratings of empathy and perspective taking for SH will be higher for participants who engage in SH empathy training compared to participants who engage in standard SH training or who are not trained.

## 3. Study 2

In Study 2, we refined our approach by comparing the SH empathy training to more realistic alternatives—a standard compliance-based SH training and a no-training control group. This study aimed to determine whether the empathy module produced greater gains in empathy, perspective taking, and bystander intentions than traditional or absent training. Participants again completed online modules.

### 3.1. Study 2 Methods

#### 3.1.1. Participants

A total of 268 people participated in Study 2. Participants were recruited from the CloudResearch Connect platform and were required to be 18 years or older and reside in the United States. To prevent overlap, participants who completed Study 1 were automatically blocked from Study 2 through CloudResearch screening, resulting in no duplicate participants across studies. Seventeen participants were eliminated for not completing at least 50% of the items, two more were eliminated for missing an attention check item, and one was eliminated for answering fewer than four of seven quiz questions correctly in the standard training condition. Of the remaining participants (*n* = 248), 58% (*n* = 144) were White, 16% (*n* = 40) were Asian, 15% (*n* = 37) were Black, 13% (*n* = 32) identified as Hispanic/Latino/a, 3% (*n* = 7) were American Indian, and 3% (*n* = 7) identified with more than one race. The majority identified their gender as “man” (53%, *n* = 131), with 45% (*n* = 112) as “woman”, and 1% (*n* = 2) as “gender as non-binary.” The average age in this study was 39.33 years old (*SD* = 13.09). Most (49%, *n* = 122) were employed full time for pay; 19% (*n* = 47) were not employed, 13% (*n* = 32) identified as a seasonal or “gig” worker, 12% (*n* = 30) were employed part-time, and 7% (*n* = 17) provided another employment status, such as retired, homemaker, and self-employed. Most (37%, *n* = 92) held a bachelor’s degree, 13% (*n* = 32) held a master’s degree, 12% (*n* = 30) held an associate’s degree, 12% (*n* = 30) held a high school or equivalent, and 2% (*n* = 5) held a professional degree. Based on data voluntarily supplied to CloudResearch (*n* = 251, see footnote 1), 8% (*n* = 20) had an occupation in information technology, 7% (*n* = 18) in STEM, 7% (*n* = 17) in education and training, 7% (*n* = 17) in medicine, and less than 6% in other occupations. There were 21% (*n* = 52) who listed “other,” and 4% (*n* = 11) who said they were retired. Of those who reported relationship status, 42% (*n* = 105) were single, 27% (*n* = 69) were married, 14% (*n* = 35) were in a relationship, and 8% (*n* = 21) were divorced. Less than 3% each listed some other status.

#### 3.1.2. Measures

In Study 2, we retained several of the measures used in Study 1 to maintain consistency and allow for meaningful comparison across studies. Specifically, we again assessed empathic concern and empathic distress (Batson et al., 1997), perspective taking for sexual harassment (PTSH; Ventura et al., 2021), and bystander intervention intentions (Liang & Park, 2022). These instruments continued to capture participants’ empathy, as well as their self-reported likelihood of intervening in harassment situations. However, based on findings from Study 1, where no significant group differences emerged, we sought to refine our measurement approach to better capture subtle changes in empathy and control for potential response biases. Therefore, in Study 2, we added the Measure of State Empathy (MSE; Powell & Roberts, 2017) to provide a more sensitive, multidimensional assessment of empathy specific to SH victims, and the Behavioral Inventory of Desirable Responding (BIDR-6; Bobbio & Manganelli, 2011) to account for socially desirable responding. These additions allowed for a more accurate evaluation of the training’s effects and helped clarify whether empathy gains were genuine or inflated by social desirability tendencies.

Socially desirable responding was measured with the Behavioral Inventory of Desirable Responding, short version (BIDR-6; Bobbio & Manganelli, 2011), which is a 16-item scale measuring two forms of desirable responding. Impression management (IM, 8 items) is an overt strategy to make good impressions on others, e.g., “I always obey laws, even if I’m unlikely to get caught.” *α* = 0.79. Self-deception enhancement (SDE, 8 items) is a less consciously aware belief that one is honest but responds socially appropriately to protect their self-esteem, e.g., “My first impressions of people usually turn out to be right.” *α* = 0.73. Responses are recorded on scales ranging from 1 (strongly disagree) to 5 (strongly agree).

The Measure of State Empathy (MSE; Powell & Roberts, 2017) is an adapted 9-item measure collective measuring cognitive empathy or understanding the way another person feels, e.g., “I understand how sexual harassment victims feel;” affective empathy or feeling the way another person feels, e.g., “I feel the same way that sexual harassment victims feel;” and compassionate empathy or feeling sympathy or concern for others, e.g., “I have feelings of concern for sexual harassment victims.” The respondents rated the extent to which they felt each statement was true for them and ranged from 1 (not at all) to 5 (entirely). We averaged all items to create a composite state empathy measure (MSE). *α* = 0.93.

Perspective Taking for Sexual Harassment (PTSH), empathic concern, empathic distress, and bystander intentions were measured with the scales described in Study 1. Reliabilities (*α*) for these measures in Study 2 were 0.87, 0.87, 0.81, and 0.90, respectively.

#### 3.1.3. Procedure

The study design was an online randomized experiment with post-tests only. The survey was accessed through the CloudResearch Connect platform which provided a link to a Qualtrics survey which contained all survey measures and training components. After reading the informed consent statement and agreeing to participate, participants watched a brief video explaining that we were testing a novel training program. Next, they completed the BIDR scales and then they were subsequently randomized into one of three conditions: SH empathy training (*n* = 74, 46% women, *M_age_* = 41), standard SH training (*n* = 87, 49% women, *M_age_* = 39), and a waitlist control condition (*n* = 87, 39% women, *M_age_* = 38).

The SH empathy training was the same as described in Study 1. For the standard, SH training condition, we followed a template of sexual harassment training that has been implemented at our institution, which was developed by our equal employment opportunity compliance office with input from legal counsel. The last author has also reviewed training programs at many institutions throughout her career—both in education sectors and private industry and agrees that our institution’s SH training is typical of that found in other industries. We modified our institution’s training program from online-video-based training program to a text-based training program so that it matched the modality of the other conditions. Information was provided regarding definitions of different forms of SH (based on the E.E.O.C., 1997), how to set boundaries, and how to understand power differentials. After reading each module, participants would be asked a question about what they had read to test their knowledge retention (which was used as an attention check). In both conditions, participants completed empathic concern, empathic distress, PTSH, and MSE measures in random order, and then completed the bystander intentions scale and a standard demographic questionnaire.

For the waitlist control condition, participants did not receive any training and were directed to complete the measures of empathy, perspective taking, bystander intentions, and demographic questions described above. All participants read a debriefing statement describing the true nature of the study and providing links to resources where they could report concerns of SH. Participants in the SH empathy training condition and the standard SH training condition were compensated with $2.50 in their CloudResearch accounts; whereas those in the waitlist control condition (which was considerably shorter) were compensated with $1.25 in the CloudResearch account. The study was approved by our university’s IRB board as an exempt protocol.

### 3.2. Study 2 Results

#### 3.2.1. Descriptive Statistics and Initial Hypothesis Testing

Eliminating outliers that were 2 *SD* above the mean, the average duration for completing the SH empathy training condition study was 21.7 min (*SD* = 9.16). Study 2 had more measures than Study 1, which may account for the longer duration. Nonetheless, this duration confirms that our empathy training program was brief.

Table 2 presents the means, standard deviations, one-way ANOVA results, and intercorrelations among the study variables. With one exception, the social desirability scales were uncorrelated with the empathy measures (except that SDE was negatively correlated with empathic concern) or with bystander intentions. Therefore, we did not conduct our remaining analyses with these measures as control variables. There were significant differences on MSE and PTSH by study condition, with those in the SH empathy training condition expressing higher ratings of state empathy (MSE) and perspective taking (PTSH) than those in either the standard SH training condition or the wait-list control condition. There was a marginally significant effect of study condition on empathic distress with those in the SH empathy training condition expressing higher empathic distress than those in the standard SH training condition (but not different from those in the wait-list control condition). The pattern of mean differences on MSE and PTSH measures provides support for modified hypothesis 1. The empathy and perspective taking measures were significantly correlated with bystander intentions, supporting hypothesis 2, which permitted testing for mediation effects of training conditions on bystander intentions through MSE and PTSH in a parallel mediation model.

#### 3.2.2. Mediation Analyses

**Figure 1:**
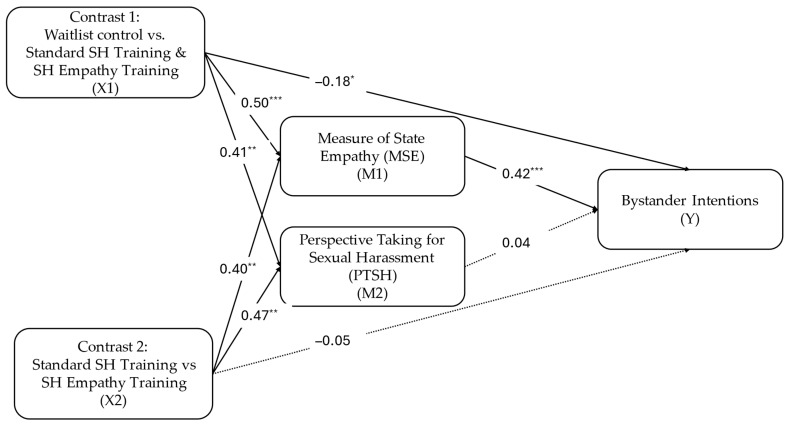
Study 2: Indirect effects of study conditions on bystander intentions through state empathy (MSE) and perspective taking (PTSH). Note: path values are unstandardized beta weights; * *p* < 0.05; ** *p* < 0.01; *** *p* < 0.001.

To test hypothesis 3, that empathy and perspective taking would mediate the relationship between training conditions, we conducted a parallel mediation analysis with Hayes’ PROCESS macro version 4.2 for SPSS (Hayes, 2022). The three training conditions were represented with a set of Helmert contrasts where contrast 1 (X1) compared the wait list control condition to the standard SH training and SH empathy training conditions combined; and contrast 2 (X2) compared the standard training to the SH empathy training. Indirect effects are assessed with bias-corrected bootstrapping with 5000 resamples, producing 95% confidence intervals around the point estimate. The statistical output for the mediation analyses is presented in Table 3 and depicted in Figure 1. Addressing hypothesis 1 (modified), the effect of the contrasts X1 and X2 on each proposed mediator were statistically significant and positive, indicating that both the standard training and the empathy training produced higher scores on both empathy (MSE) and perspective taking (PTSH; X1 contrast), and that empathy training produced higher scores on these measures than the standard training condition (X2 contrast). Furthermore, controlling for PTSH, MSE was significantly positively associated with bystander intentions; however, controlling for MSE, PTSH was not (partially supporting hypothesis 2). MSE and PTSH were highly correlated (*r* = 84, *p* < 0.001) and both were correlated with bystander intervention intentions (see Table 2); however, once MSE was accounted for, PTSH added no incremental variance in the prediction of bystander intervention intentions. Consequently, the indirect effects of both the X1 and X2 contrasts on bystander intervention intentions through MSE were significant; whereas the indirect effects through PTSH were not (see Table 3). Hence, hypothesis 3 was supported for MSE as a mediator but not for PTSH.

There was a significant negative conditional direct effect of contrast X1 on bystander intentions, suggesting that when MSE and PTSH are controlled, the waitlist condition produced higher bystander intentions than either of the training conditions (See Table 3 and Figure 1). We do not have an explanation for this finding other than that it may be an alpha error since it was the only significant direct effect in either analysis. However, this non-hypothesized finding may suggest a suppression effect, such that controlling for third variable (e.g., state empathy and perspective taking) strengthens the association between a predictor (e.g., X1 contrast) with an outcome variable (e.g., bystander intentions; MacKinnon et al., 2000). We address this speculation more in the discussion section.

#### 3.2.3. Gender Differences and Moderation Effects

To test gender differences on the study variables, we conducted independent sample *t*-tests. There were significant gender differences on MSE, *t* = 2.40, *p* = 0.017, Cohen’s *d* = 0.31, with women having higher empathy ratings (*M* = 3.40, *SD* = 0.99) than men (*M* = 3.10, *SD* = 0.95); and on PTSH, *t* = 3.89, *p* < 0.001, Cohen’s *d* = 0.50, with women having higher perspective taking ratings (*M* = 3.34, *SD* = 1.05) than men (*M* = 2.82, *SD* = 1.02). To test whether gender moderated the mediated effects of study condition on bystander intentions through MSE and PTSH, we conducted moderated mediation analyses with Hayes’ PROCESS macro for SPSS v. 4.2 (Hayes, 2022). Gender did not moderate the effect of contrast 1 on MSE, *b* = −0.22 (*SE* = 0.25), *p* = 0.387, nor the effect of contrast 2 on MSE, *b* = 0.48 (*SE* = 0.30), *p* = 0.113. Likewise, gender did not moderate the effect of contrast 1 on PTSH, *b* = −0.14 (*SE* = 0.27), *p* = 0.615 or contrast 2 on PTSH, *b* = 0.38 (*SE* = 0.32), *p* = 0.244. Gender also did not moderate the conditional direct effect of contrast 1 on bystander intentions, *b* = 0.10 (*SE* = 0.17), *p* = 0.574; or the conditional direct effect of contrast 2 on bystander intentions, *b* = −0.10 (*SE* = 0.21), *p* = 0.633. The positive indirect effect of contrasts 1 and 2, noted above, were statistically the same for women and men; although for men the indirect effect of contrast 1 through MSE was negative and nonsignificant, *b* = −0.09 (*SE* = 0.09), 95% CI: [−0.09, 0.28]. The nonsignificant indirect effects of contrasts 1 and 2 on bystander intervention intentions through PTSH were statistically the same for women and men. These findings suggest that while women express more empathy and perspective taking for SH victims than men, the effectiveness of the SH empathy training on bystander intentions through MSE is equally effective for women and men.

### 3.3. Study 2 Discussion

Study 2 found evidence that brief SH empathy training can effectively increase bystander intentions through its effect on empathy (MSE) and that the training is equally effective for women and men. The more ecologically valid comparisons between SH empathy training with a more standard form of compliance-based SH training and a non-training condition permitted a more realistic assessment of training comparison than did Study 1. To allay our concern from Study 1, measures of social desirability were not associated with our mediating or outcome measures. Furthermore, newer measures of empathy (MSE) and perspective taking (PTSH) were more sensitive than older measures of empathy (empathic concern and empathic distress, Batson et al., 1997) to the effects of empathy training. The empathic concern and empathic distress measures used in the current research may not have cognitively engaged participants in their understanding of empathic feelings and perspectives toward the victim. Participants simply identified emotions they were feeling toward the target. More recent discussions of empathy regard it as a multi-faceted construct that includes affective empathy (emotional resonance) and cognitive empathy (perspective taking; Decety & Jackson, 2004). Sympathy is a related construct that includes feelings of sorrow or sadness for the empathy target (Eisenberg & Miller, 1987). All three components—affective empathy, cognitive empathy, and sympathy—composed the MSE scale. Although both MSE and PTSH were significantly correlated with empathic concern and empathic distress, which provides evidence for construct validity for these measures, they more clearly reflect the components of resonance, perspective taking, and sympathy. Moreover, items in these scales are more detailed sentences or phrases about their responses to the victim, which may have allowed participants to engage more deeply with the measures. MSE and PTSH were very highly correlated, though, which suggests that they may be measuring the same construct, particularly since cognitive empathy, also known as perspective taking, was a component of both measures. Therefore, the use of both MSE and PTSH in future research may not be necessary.

Controlling for MSE and PTSH participants in the waitlist control condition expressed higher intentions to engage in bystander intervention behaviors (see Figure 1). We do not have a ready explanation for this finding. This may be evidence of a suppression effect, such that when the empathic impact of sexual harassment training is accounted for there may be pressure upon non-trained participants to provide a socially desirable response. However, measures of socially desirable responding, as individual difference variables (IM and SDE), were not associated with bystander intentions; hence, socially desirable responding may have been state dependent (see Krumpal, 2013). Future researchers may want to explore situational influences on socially desirable responding in the context of SH training research.

## 4. General Discussion

The present research tested whether a brief, online-capable empathy training module focused on SH could enhance bystander intervention intentions through its effects on empathy and perspective taking. Across two studies, we found partial support for our hypotheses. Study 1 revealed positive associations between empathy and bystander intentions but no significant differences across training conditions. In Study 2, however, SH empathy training was more effective than both standard compliance-based SH training and a no-training control in increasing state empathy and perspective taking, for which MSE, in turn, mediated increases in bystander intervention intentions. These findings provide new evidence for the role of empathy as a mechanism in SH prevention training and point to the value of incorporating empathic perspective-taking exercises focusing on personally relevant episodes of harassment into existing compliance-oriented approaches.

### 4.1. Theoretical Implications

Our findings contribute to ongoing debates about the role of empathy in fostering prosocial responses to interpersonal harm. Consistent with theoretical frameworks that emphasize empathy and perspective taking as antecedents of moral sensitivity and prosocial action (Batson et al., 1997; Decety & Jackson, 2004; Eisenberg & Eggum, 2009), the current results highlight that even brief interventions can effectively shift psychological processes relevant to bystander intervention, specifically empathy and perspective taking. The significant mediation results from Study 2 support models that conceptualize empathy as a constellation of affect (feeling SH victims’ feelings), perspective taking (understanding SH victims’ feelings), and compassion (feeling sympathetic toward SH victims) encourages intervention when witnessing misconduct (Liang & Park, 2022). Moreover, the high correlation between state empathy (MSE) and perspective taking (PTSH) suggests that these constructs may converge in contexts of SH training, raising theoretical questions about whether affective and cognitive components of empathy function synergistically rather than distinctly in workplace interventions.

### 4.2. Practical Implications

From a practical standpoint, our findings indicate that brief empathy training modules can be feasibly integrated into organizational SH prevention programs. Traditional compliance-based training, while necessary for legal and policy reasons, often shows limited effectiveness in shifting attitudes or motivating action (Dobbin & Kalev, 2019; Roehling et al., 2022). Guided imagery of actual accounts of SH from a first-person perspective and structured perspective-taking and reflection exercises can be easily embedded into existing training which may enhance participants’ readiness to act as proactive bystanders. Importantly, gender did not moderate the indirect effects of training on bystander intentions, suggesting that well-designed empathy training can be equally effective across men and women despite baseline gender differences in empathy (Christov-Moore et al., 2014); however, empathy training did not close the gap between women and men on bystander intentions. Nonetheless, organizations seeking scalable, evidence-based tools for SH prevention may benefit from supplementing compliance training with targeted empathy-building exercises. However, we note that our research did not test empathy training as a supplement to traditional compliance training—instead we compared it to traditional training. Therefore, future research should test its incremental value over traditional, compliance-based training.

### 4.3. Limitations

Several limitations should be acknowledged. First, our reliance on self-reported empathy and bystander intentions raises concerns about social desirability and demand characteristics, despite the inclusion of impression management and self-deception measures in Study 2. Future work should examine behavioral outcomes, such as actual bystander actions in simulated or naturalistic contexts (Kettrey & Marx, 2019). Second, although the brief nature of our intervention makes it practical, it is unclear whether effects persist over time. Longitudinal assessments are necessary to determine durability. Third, the strong overlap between state empathy and perspective taking raises questions about constructing distinctiveness in this context, warranting further psychometric refinement. Finally, although our samples were diverse in terms of race and gender, they were limited to U.S. adults recruited online, which may constrain generalizability to organizational or academic populations where SH training is implemented.

### 4.4. Future Directions

Future research should extend this work in several ways. First, researchers should test the long-term effects of empathy training, examining whether increases in empathy and bystander intentions translate into actual intervention behaviors over time. Longitudinal research could also examine how emotional empathy and perspective taking work synergistically. For example, does empathy give rise to perspective taking which gives rise to behavior or behavioral intentions, or does perspective taking give rise to empathy to invoke action? Open-ended prompts to the personal reflection exercises could be searched for phrases such as “this training helped me feel what SH victims are feeling; now I understand what they are going through.” Rigorous longitudinal designs could also test the temporal ordering of empathy and perspective taking. Second, research should examine other important training outcomes, such as the reduction in myths about SH (Lonsway et al., 2008) and the reduction in intentions to engage in SH (Grabowski et al., 2022). Third, examining intersectional differences in responses to empathy training (e.g., across racial/ethnic or sexual minority groups) could illuminate whether tailored approaches are necessary to maximize effectiveness (Clancy et al., 2014). Finally, integrating empathy training within broader psychosocial safety climate interventions may provide synergistic benefits in reducing harassment and fostering organizational cultures of respect (Amoadu et al., 2024).

## 5. Conclusions

Our findings demonstrate that brief empathy training can enhance state empathy and perspective taking, which in turn increases bystander intervention intentions in SH contexts. While compliance-based training remains an essential organizational tool, its impact can be strengthened through the integration of empathy-based modules. By cultivating perspective-taking and empathy, organizations may not only reduce the prevalence of SH but also empower employees to act as allies. Continued research on the mechanisms, durability, and contextual applicability of empathy training will be critical for building comprehensive, evidence-based approaches to SH prevention.

## Figures and Tables

**Table 1:** Study 1 Means and *SD*s by training condition, one-way ANOVA results, and intercorrelations among study variables.

	Training Condition—Mean (SD)				Correlations		
	Time Management	Burglary Empathy	SH Empathy	F (*p*) df = 2, 259	(a)	(b)	(c)
(a) Bystan	4.08 (0.62)	4.06 (0.69)	4.13 (0.66)	0.28 (*p* = 0.255)			
(b) EmpCon	5.49 (0.90)	5.35 (0.90)	5.65 (0.81)	1.81 (*p* = 0.166)	**0.48**		
(c) EmpDiss	4.12 (1.20)	4.46 (1.25)	4.46 (1.33)	2.12 (*p* = 0.122)	**0.23**	**0.21**	
(d) Oneness	3.86 (1.80)	3.59 (1.61)	3.99 (1.71)	1.23 (*p* = 0.294)	**0.35**	**0.24**	0.11

Notes: Bold-faced correlations are significant, *p* < 0.001. SH = sexual harassment. Bystan = bystander intervention intentions; EmpCon = empathic concern; EmpDiss = empathic distress.

**Table 2:** Study 2 Means and *SD*s by training condition, one-way ANOVA results, and intercorrelations among study variables.

Variable	Study Condition—Means (SD)				Correlations					
	Waitlist Control	Standard SH Training	SH Empathy Training	F (*p*) df = 2, 245	(a)	(b)	(c)	(d)	(e)	(f)
(a) ByStan	4.03 (0.83)_a_	4.01 (0.74)_a_	4.15 (0.70)_a_	0.71 (*p* = 0.495)						
(b) EmpCon	5.42 (1.12)_a_	5.40 (1.11)_a_	5.47 (0.95)_a_	0.08 (*p* = 0.921)	**0.54**					
(c) EmpDiss	3.91 (1.44)_a,b_	3.73 (1.44)_a_	4.25 (1.26)_b_	2.78 (*p* = 0.064)	**0.29**	**0.27**				
(d) MSE	2.94 (0.94)_a_	3.24 (1.03)_a_	3.64 (0.81)_b_	11.16 (*p* < 0.001)	**0.55**	**0.53**	**0.16**			
(e) PTSH	2.81 (1.12)_a_	2.99 (1.03)_a_	3.46 (0.92)_b_	8.34 (*p* < 0.001)	**0.48**	**0.45**	**0.15**	**0.84**		
(f) IM	3.21 (1.10)_a_	3.19 (0.97)_a_	3.31 (0.94)_a_	0.28 (*p* = 0.753)	0.06	−0.03	0.04	−0.02	−0.04	
(g) SDE	4.02 (0.94)_a_	4.02 (0.71)_a_	3.97 (0.68)_a_	0.09 (*p* = 0.911)	−0.02	**−0.16**	0.00	−0.11	−0.10	**0.39**

Notes: Bold-faced correlations are significant, *p* < 0.01 to *p* < 0.001. Per row (variable), means that are significantly different (*p* < 0.05) from others are denoted with different subscripts assessed with a Tukey post hoc test. SH = sexual harassment; ByStan = bystander intervention intentions; EmpCon = empathic concern; EmpDiss = empathic distress; MSE = measure of state empathy, IM = impression management; SDE = self-deception enhancement.

**Table 3:** Study 2, parallel mediation analyses of the effects of training conditions on bystander intervention intentions through MSE and PTSH, *n* = 248.

Predictor/Row Information	Outcome Variable								
	MSE (M1)			PTSH (M2)			Bystander Intentions (Y)		
	*B* (*SE*)	*p*	95% CI	*B* (*SE*)	*p*	95% CI	*B* (*SE*)	*p*	95% CI
Contrast 1 (X1)	0.50 (0.12)	<0.001	0.25, 0.75	0.41 (0.14)	0.003	0.14, 0.68	−0.18 (0.09)	0.043	−0.35, −0.01
Contrast 2 (X2)	0.40 (0.15)	0.008	0.11, 0.69	0.47 (0.16)	0.004	0.15, 0.79	−0.05 (0.10)	0.603	−0.25, 0.15
MSE	–			–			0.42 (0.08)	<0.001	0.27, 0.57
PTSH	–			–			0.04 (0.07)	0.591	−0.10, 0.18
Indirect Bootstrap estimates X1→M1/M2→Y	0.21 (0.07)	95% CI: 0.09, 0.36		0.02 (0.04)	95% CI: −0.06, 0.09				
X2→M1/M2→Y	0.17 (0.07)	95% CI: 0.05, 0.33		0.02 (0.04)	95% CI: −0.06, 0.10				
Model Summary	*R*^2^ = 0.08 *F*(2, 245) = 11.16, *p* < 0.001			*R*^2^ = 0.06 *F*(2, 245) = 8.34, *p* < 0.001			*R*^2^ = 0.32 *F*(4, 243) = 28.61, *p* < 0.001		

Notes: Contrast 1 (X1) compares the waitlist control condition (coded −0.67) to the standard training and empathy training conditions (each coded 0.33). Contrast 2 (X2) compares the standard training condition (coded −0.50) to the empathy training condition (coded 0.50). MSE = Measure of State Empathy; PTSH = Perspective Taking for Sexual Harassment.

## Data Availability

Datasets are available in the Supplementary Materials.

## Note

1 Data from CloudResearch could not be matched case by case to the dataset downloaded from Qualtrics because we did not collect the participant ID number for confidentiality purposes. The CloudResearch data set, therefore, contains participants that we eliminated from our Qualtrics file.

## Supplementary Materials

The following supporting information can be downloaded at https://www.mdpi.com/article/10.3390/bs16020227/s1: (1) SPSS datasets; (2) IRB materials (informed consent statements and IRB approval statements; (3) copies of all measures.

## Abbreviations

The following abbreviations are used in this manuscript:

BIDR	Balanced Inventory of Desirable Responding
HMD	Head Mounted Display
IM	Impression Management
MSE	Measure of State Empathy
PTSH	Perspective Taking for Sexual Harassment
SDE	Self-deception Enhancement
SH	Sexual Harassment
SMART	Specific, measurable, achievable, relevant, time-bound
STEM	Science, Technology, Engineering, and Mathematics
