# Supplementary material for: Can Brief Empathy Training Increase Sexual Harassment Bystander Intervention Intentions?
Source: Behav Sci (Basel). 2026 Feb 4;16(2):227. doi: 10.3390/bs16020227 (PMC12938127; doi:10.3390/bs16020227)
Supplement: Supplementary file 1 [file behavsci-16-00227-s001.zip › IRB Approval 27033_Study 1.pdf]

INDIANA UNIVERSITY

APPROVAL LETTER

To: Stockdale, Peggy

Protocol #: 27033

Protocol Title: Empathy Training - Abbreviated

Type of Submission: Initial

Level of Review: Exempt

Approval Date: Friday, April 25th 2025

Expiration Date: no date provided

**If Expiration Date = "No date provided," this research does not require annual renewal; thus there is no expiration date.*

The Indiana University HRPP approved the above-referenced submission. Conduct of this study is subject to the IU HRPP Policies, as applicable.

Additional Notes:

This research is exempt under the following category:- Category 2(ii)

Documents approved with this submission:

Attachments

Study Information Sheet	Study Information Sheet - Study Information Sheet_Abbreviated Empathy Training Pilot.docx
Recruitment Materials	Recruitment statement_Abbreviated Empathy Training Pilot.docx
Data Collection Instrument	Data Collection Instrument_Abbreviated Empathy Training_Pilot.pdf

You should retain a copy of this letter and all associated approved study documents in your research records.

If you have any questions or require further information, please contact the HRPP via email at irb@iu.edu or via phone at (317) 274-8289.
