# Supplementary material for: Can Brief Empathy Training Increase Sexual Harassment Bystander Intervention Intentions?
Source: Behav Sci (Basel). 2026 Feb 4;16(2):227. doi: 10.3390/bs16020227 (PMC12938127; doi:10.3390/bs16020227)
Supplement: Supplementary file 1 [file behavsci-16-00227-s001.zip › Other trainings.pdf]

Other Training Materials

Contents

Burglary Empathy Training 1

Time Management Training 3

Standard, compliance-based training..5

Burglary Empathy Training

Think about a story of **someone other than you,** who was burglarized (robbed)**.**This may be someone you know or someone you've heard about, e.g., in the media. Take some time to think about the details. **Where did it happen? When did it happen? Who else was involved? How did the experience affect them?** In the space below write down all the details in 15 or more words.

Now, think about this same occasion, but **imagine that YOU are the person who experienced this burglary.** Describe in first-person and present-tense, as the person in this situation. For example, "I am walking to my car after meeting friends for dinner when ...."

What are you experiencing? Who is with you? Where is it occurring? How is the experience affecting you? Provide as much detail as you can, paying special attention to your thoughts and feelings as the victim throughout the entire narrative.

Continuing to take the perspective of a person who was burglarized, in the first person, think about everything that happened to you and how those experiences affected you immediately following the burglary. **Identify as many of the short-term consequences that resulted from those events**, using 10 or more words**.** These consequences may be emotional, psychological, professional/academic, social, etc. These consequences may be emotional, psychological, professional/academic, social, etc. For example, you might say, shortly after the harassment, I was ... (I felt ..., I experienced...

Now, consider the more lasting ramifications brought about by that experience. **Identify as many of the long-term consequences that resulted from those events**, using 10 or more words**.** These consequences may be emotional, psychological, professional/academic, social, etc. For example, you might say, several months (years?) after the harassment I felt..., I experienced... etc.)

Now it is time to reflect on your own experiences during the empathy training exercises. Please respond to the following questions.

What emotions and thoughts were evoked when you wrote about someone else's burglary experience? How might those emotions and thoughts change your attitudes and beliefs about crime? In other words, how did it feel to recount the experience and the aftermath as if it happened to you?

What did you learn from this story of burglary? What new insights regarding burglaries did you get from this story?

Time Management Training

You will engage in **time management** training focusing on goal setting.

First, **write 3 sentences** (total of 15 words or more) about habits that you or others may have that waste time. How do these habits affect your productivity and efficiency?

The next step to develop good time management habits is to state your goals. Think of a goal you want to accomplish. Use the rubric below to write a SMART goal.

- **Specific:** Think of a specific, concrete goal.
- **Measurable:** Is that goal something that you can measure; that is, can you quantify your goal? If not, reconceptualize your goal so that it is something you can measure.
- **Achievable:** Is your goal realistic and attainable. It should stretch your abilities but remain possible.
- **Relevant:** Is this goal important to you? Is it worth pursuing? If not, reconceptualize your goal to reflect important long-term objectives you want to achieve.
- **Time-bound:** Is there a sense of urgency or priority to your goal? If not, reconceptualize your goal to reflect a goal that has a deadline.

Now, state your goal, in 15 words or more, so that it is Specific, Measurable, Achievable, Relevant, and Time-bound.

Now, you will learn strategies to avoid procrastination. Think of the SMART goal you just wrote. Break down that goal into 4 tasks or sub goals that need to be completed to achieve your goal.

Of those four tasks or subgoals, which one is **Urgent and Important**? List this subgoal below.

Which is **Important but not Urgent**? Make a schedule of when you will complete this task or subgoal

Which is **Urgent but not Important**: Delegate this task to someone else

Which is not **Urgent and Not Important**. Eliminate this task

Now, we will focus on accountability

- In the space below, in 15 words or more, describe how you will hold yourself accountable to reaching your goal. Who will you **share** your goal with? What will you ask them to do to keep you accountable? How will you **reward yourself** for meeting each of the steps, stages, or subgoals to reaching your final goal?

Standard, compliance-based training

The purpose of anti-harassment training is to create a safe, nondiscriminatory environment for all employees. Sexual harassment and harassment related to personal characteristics, such as age, race/ethnicity, national origin, or religion, is prohibited by both federal and state laws.

In this training you will learn about behavior that constitutes sexual harassment, practices for maintaining harassment-free work environments, and procedures for reporting harassment should you experience, observe, or hear about other people's harassment. Please pay close attention to the material. There will be a quiz throughout the training to test your knowledge.

<page break>

What is the purpose of this training?

- ○ To help employees negotiate salaries
- ○ To prepare employees for leadership roles
- ○ To create a safe, nondiscriminatory work environment for everyone
- ○ To teach employees how to write formal complaint

<page break>

According to the law, both sexual harassment and harassment based on sex or gender are not allowed. The following information describes ***gender harassment*.**

Anyone regardless of gender, race, or background, can experience sexual or gender-based harassment. It's important to understand what these behaviors mean. Sex or gender-based harassment includes words, actions, images, or behaviors that are mean, threatening, or aggressive.

These behaviors are based on someone's sex, gender, sexual orientation, or gender identity. This kind of behavior is not allowed when it happens often or is serious enough to stop someone from fully taking part in school or work activities.

For example, if someone is repeatedly teased for not acting "manly" or "feminine" enough, or if they are left out of a group because of their sexual orientation or gender identity, that behavior is considered gender harassment and is not allowed.

<page break>

An example of gender-based harassment is:

- ○ Promising a raise to an employee who agrees to date you.
- ○ Touching another person in a sexually inappropriate way.
- ○ Asking an employee out for a date.
- ○ Repeatedly teasing an employee for not acting "manly" or "feminine" enough.

<page break>

Sexual harassment involves unwanted conduct related to sex or sexual activity, including inappropriate actions, attention, or communication where:

- An employee or employer conditioned the provision of employment, such as obtaining or losing a job, promotion, or other job-related opportunity on an individual's participation in unwelcome sexual conduct. Even if a bribe or threat of a job opportunity isn't at stake, unwelcome sexual conduct constitutes sexual harassment if it creates a hostile work environment for one or more employees.
- An employee or employer gives unwanted sexual attention to an employee such as touching, ogling, repeated requests for dates, repeated unwanted texts, email or phone calls of a sexual nature.

Unwelcome conduct is determined by a reasonable person to be so severe, pervasive, and objectively offensive that it effectively denies a person equal access to employment opportunities. Severity, pervasiveness, and objective offensiveness are evaluated based on the totality of the circumstances from the perspective of a reasonable person in the same or similar circumstances as the victim, including the context in which the alleged incident occurred and any similar, previous patterns that may be evidenced.

<page break>

An employee who asks another employee for a date and then stops asking when the recipient has said no, has committed sexual harassment.

- ○ True
- ○ False

<page break>

You may serve in a variety of roles at your place of employment. It can sometimes be challenging to clearly define and maintain appropriate boundaries with others in your work setting. This is particularly true for those of you who are close in age to your coworkers, bosses, or subordinates, since you may have encountered them in contexts outside of the

workplace, including after-work social events, work-related travel, and so forth. However, regardless of your age, as an employee how you interact with these colleagues as well as any employee you supervise does matter, especially regarding behaviors that fall within the sexual harassment policy. If you were to meet with your coworkers outside of the physical location where you work or in a different situation or context, the boundaries you set would likely look different. But as an employee, you may be in a position of power over others, which requires you to take special care not to engage in, condone, or turn a blind eye to harassment that you may or should know about.

<page break>

What factor may increase the complexity of setting boundaries in the workplace?

- ◯ Differences in educational background.
- ◯ Close age proximity or overlapping social circles outside of work.
- ◯ Having similar job descriptions.
- ◯ Wearing casual attire on Fridays.

<page break>

A power differential means having greater influence or authority over others, which can affect evaluations, promotions, or opportunities. For example, when a supervisor has the role of evaluating, supervising, or making a recommendation for an employee, the supervisor exercises a certain degree of authority over that employee. If you are a mid-level supervisor you may experience both roles -- a person who has power over others, as well as a person for whom others have power over you such as your superiors.

Your power over others may impact their performance evaluations, pay, promotions, recommendations, job assignments, special opportunities, and so forth. Similarly, your superiors can shape those opportunities for you. Even when considering colleagues at the same level as you, there may be power differentials such as popularity, seniority, and physical strength that may affect how others are treated.

<page break>

Which of the following best describes a power differential in the work place?

- ◯ A difference in physical strength between employees that results in favoritism.
- ◯ A situation where one person has greater authority or influence over another, which can impact evaluations, promotions, and opportunities.
- ◯ A policy that ensures all employees are treated equally regardless of their rank.
- ◯ A disagreement between two coworkers of equal rank.

<page break>

Best practices for maintaining appropriate boundaries with employees over whom you have power include:

- Scheduling meetings with employees in your office or in another appropriate space to hold a meeting
- Keeping doors open.
- Ensure confidentiality, especially during sensitive conversations such as performance evaluations.
- Using work-sponsored professional communication channels such as through a sanctioned work phone or regular email. Avoid communicating through social media or through employees' personal phones, such as texting.
- Maintaining professionalism and boundaries during work-related or work-sponsored social events.

Why is it important to interact with employees only during scheduled times and through official communication channels?

○ It allows you to avoid doing extra work.

○ It ensures employees do not ask for help that is not available to others.

○ It helps maintain professional boundaries and reduces the risk of inappropriate interactions.

○ It makes it easier to evaluate employees job performance.

<page break>

There are also situations where other people, such as managers and company leaders, have power over you. It is important to know and understand the boundaries surrounding you and your relationship with your superiors.

The list of inappropriate situations we discussed earlier where you are less powerful than others are important to recognize as well. Never accept sexual harassment as normal or acceptable. If you find yourself in such a situation, you should reach out for support and assistance. Remember that you have the right to a harassment-and discrimination-free work environment, and you have the right to be protected from retaliation.

Understand your organization's policy on sexual harassment and available reporting options. Typically, this includes an HR office or representative and possibly an anonymous hotline.

What should you do if you experience or witness behavior from a superior that may constitute sexual harassment?

- ○ Ignore it and hope it stops.
- ○ Confront the superior immediately in public.
- ○ Reach out for support and use available reporting options.
- ○ Resign from your position as soon as possible.

<page break>

Take away points:

- Gender and sexual harassment is illegal and against company policy.
- Avoid behaviors that might lead to harassment, even if it is unintentionally done.
- Look for signs that you or others are abusing their power over others.
- Report any suspicion of harassment to a Human Resources representative.
- Do not retaliate against someone who reports harassment or assists in a harassment investigation.
