# Supplementary material for: Can Brief Empathy Training Increase Sexual Harassment Bystander Intervention Intentions?
Source: Behav Sci (Basel). 2026 Feb 4;16(2):227. doi: 10.3390/bs16020227 (PMC12938127; doi:10.3390/bs16020227)
Supplement: Supplementary file 1 [file behavsci-16-00227-s001.zip › Sexual Harassment Empathy Training.pdf]

Sexual Harassment Empathy Training – Abbreviated

Instructions:

Prompt 1: Think about a story of **someone other than you** who has experienced sexual harassment. This may be someone you know or someone you've heard about, e.g., in the media. Take some time to think about the details. **What happened, who did it, where did it occur, how did it affect them?**

Prompt 2: Now, think about this same incident, **but imagine that YOU are the person who experienced this sexual harassment**. Describe the harassment in **first-person** and **present-tense**, as the person experiencing sexual harassment that you described in your answer to the question above. For example, "I am attending a meeting for work, and "Joe" comes up to me and says..."

What are you experiencing? Who was the perpetrator (you can use a false name)? Where is it occurring? Provide as much detail as you can, paying special attention to your thoughts and feelings as the victim throughout the entire narrative.

Prompt 3:

Continuing to take the perspective of a person experiencing sexual harassment, in the first person, think about everything that happened to you and how those experiences affected you immediately following the incident. **Identify as many of the short-term consequences that resulted from those events,** using 10 or more words. These consequences may be emotional, psychological, professional/academic, social, etc. For example, you might say, shortly after the harassment, I was ... (I felt ..., I experienced...)

Prompt 4: Now, consider the more lasting ramifications brought about by those experiences. **Identify as many of the long-term consequences that resulted from those events**, using 10 or more words**.** These consequences may be emotional, psychological, professional/academic, social, etc. For example, you might say, several months (years?) after the harassment I felt…, I experienced… etc.)

Prompt 5: Now it is time to reflect on **your own experiences** during the empathy training exercises. Please respond to the following questions.

What emotions and thoughts were evoked when you wrote about someone else's sexual harassment experiences? In other words, how did it feel to recount the experience and the aftermath as if it happened to you?

Prompt 6: What did you learn from this story and exercise with sexual harassment? What new insights regarding sexual harassment did you get from this story?

Prompt 7: On the basis of this training, describe what actions you will take or avoid taking, in the context of workplace sexual harassment.
