# Supplementary material for: Can Brief Empathy Training Increase Sexual Harassment Bystander Intervention Intentions?
Source: Behav Sci (Basel). 2026 Feb 4;16(2):227. doi: 10.3390/bs16020227 (PMC12938127; doi:10.3390/bs16020227)
Supplement: Supplementary file 1 [file behavsci-16-00227-s001.zip › Study Information Sheet_Abbreviated Empathy Training_Study 1.pdf]

INDIANA UNIVERSITY STUDY INFORMATION SHEET FOR RESEARCH

Indiana University Research Study #27033

You are being asked to participate in a research study. Scientists do research to answer important questions that might help change or improve the way we do things in the future. This document will give you information about the study to help you decide whether you want to participate. Please read this form, and ask any questions you have, before agreeing to be in the study.
All research is voluntary. You can choose not to take part in this study. If you decide to participate, you can change your mind later and leave the study at any time. You will not be penalized or lose any benefits if you decide not to participate or choose to leave the study later.
This research is intended for individuals 18 years of age or older. If you are under the age of 18, do not complete the survey.

This research is for residents of the United States. If you are not a U.S. resident, do not complete the survey.

The purpose of this study is to evaluate different methods that might be used in a corporate or community organization setting. This research will help determine whether our training method may be effective and incorporated into longer, more comprehensive training programs.

We are asking you if you want to be in this study because you are an adult in the U.S. and you signed up to participate through CloudResearch's Connect program. The study is being conducted by Dr. Peggy Stockdale, Professor of Psychology at Indiana University Indianapolis and by Tristan Barta and Zachary Piper, who are students at this university.

If you agree to be in the study, you will do the following things: You will be randomly assigned to one of three study conditions. Depending on the condition you may be asked to recall an experience or think about your work habits. You will be asked to describe the experience or habits and then answer additional prompts. Afterwards, you will complete a few brief questionnaires, including a demographic question. The entire study takes about 20 minutes to complete.

All study interactions and survey measures will be conducted in an online environment. You are asked to be in a quiet location using a laptop or desktop computer (not a cell phone, IPad, or other mobile device) and to complete the study in one session. There is a brief video at the beginning of the study, so if you're not in a private location, we suggest that you put on earphones.

Before agreeing to participate, please consider the risks and potential benefits of taking part in this study. There is a risk that observing the stories portrayed in the study may be disturbing to some people. You may be uncomfortable while answering the survey questions. While completing the survey, you can skip any questions that make you uncomfortable or that you do not want to answer.

There is also some risk to loss of confidentiality. There is a risk that someone outside the study team could get access to your research information from this study. More information about how we will protect your information to reduce this risk is below.

We don't think you will have any personal benefits from taking part in this study, but we hope to learn things that will help researchers in the future.

You will be paid $3.00 for participating in this study through CloudResearch.

We will protect your information and make every effort to keep your personal information confidential, but we cannot guarantee absolute confidentiality. No information which could identify you will be shared in publications about this study. The researchers will detach all personal information from your research record (e.g., geolocation, CloudResearch ID) and save it in a separate, encrypted and password protected file that cannot be linked to your study data.

Your personal information may be shared outside the research study if required by law. We also may need to share your research records with other groups for quality assurance or data analysis. These groups include the Indiana University Institutional Review Board or its designees, and state or federal agencies who may need to access the research records (as allowed by law).

If you have questions about the study or encounter a problem with the research, contact the researcher, Dr. Peggy Stockdale at 317-278-3838 or pstockda@iu.edu.

For questions about your rights as a research participant, to discuss problems, complaints, or concerns about a research study, or to obtain information or to offer input, please contact the IU Human Research Protection Program office at 800-696-2949 or at irb@iu.edu.

Thank you for agreeing to participate in our research. Before you begin, please note that the data you provide may be collected and used by Cloud as per its privacy agreement. Additionally, this research is for residents of the United States over the age of 18*; if you are not a resident of the United States and/or under the age of 18, please do not complete this survey.
