# Supplementary material for: Can Brief Empathy Training Increase Sexual Harassment Bystander Intervention Intentions?
Source: Behav Sci (Basel). 2026 Feb 4;16(2):227. doi: 10.3390/bs16020227 (PMC12938127; doi:10.3390/bs16020227)
Supplement: Supplementary file 1 [file behavsci-16-00227-s001.zip › Study Measures.pdf]

Study measures

1. Empathic Concern and Distress
    a. Instructions: For each emotion listed below, please select the statement that describes your feelings toward people who have experienced sexual harassment. Do not worry if you are not feeling many of these emotions; only a few may apply. Be sure to select a response for each item.
    b. Scale

| Strongly disagree (1) | Disagree (2) | Somewhat disagree (3) | Neither agree nor disagree (4) | Somewhat agree (5) | Agree (6) | Strongly agree (7) |
|---|---|---|---|---|---|---|

    c. Items:
        i. Empathic concern
            1. Grieved
            2. Moved
            3. Sympathetic
            4. Compassionate
            5. Tenderhearted
            6. Warm
            7. Softhearted
            8. Concerned
        ii. Empathic distress
            1. Shocked
            2. Offended
            3. Irritated
            4. Upset
            5. Disturbed
            6. Distressded
            7. Sad

2. Perspective Taking for Sexual Harassment
    a. Instructions Choose the response that corresponds to the extent to which you experienced each situation described in the statements below.
    b. Scale

| Not at all (1) | a little bit (2) | Moderate extent (3) | To much extent (4) | Totally (5) |
|---|---|---|---|---|

    c. Items:
        i. To what extent do you identify with a person who claims to have experienced sexual harassment?
        ii. Do you ever imagine how you would act if you were a victim of sexual harassment?
        iii. To what extent do you see yourself from the perspective of a person who claims to have been sexually harassed?
        iv. How emotionally affected do you feel when someone shares their experience of being sexually harassed?
        v. To what extent do you worry about what happens to people who claim to have been sexually harassed?
        vi. To what extent do you understand the emotions of sexual harassment victims while they are being harassed?

3. Oneness
   a. Instructions: Which image symbolizes your sense of oneness with people who claim to be victims of sexual harassment?

| | | | | | | |
|---|---|---|---|---|---|---|
| 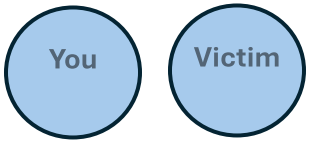  | 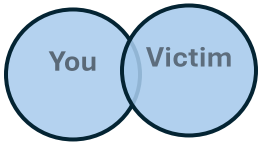  | 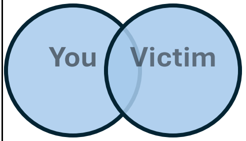  | 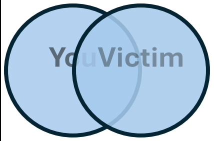  | 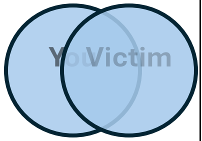  | 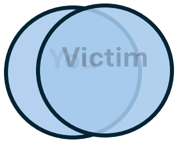  | 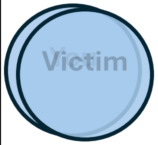  |
| 1 | 2 | 3 | 4 | 5 | 6 | 7 |

4. Measure of State Empathy
    a. Instructions: On the following scale, rate the extent to which you feel each statement is true for you. Be sure to select a response for each item.
    b. Scale

| Not at all (1) | A little bit (2) | Moderately (3) | To much extent (4) | Entirely (5) |
|---|---|---|---|---|

    c. Items
        i. I understand how harassment victims are feeling.
        ii. I know how harassment victims feel emotionally.
        iii. I can identify the feelings that sexual harassment victims are having.
        iv. Sexual harassment victim's feelings transfer to me.
        v. I feel the same way that sexual harassment victims feel.
        vi. I experience the same emotions as sexual harassment victims.
        vii. I have feelings of concern for sexual harassment victims.
        viii. I experience sympathy for sexual harassment victims.
        ix. I feel a sense of compassion for sexual harassment victims

5. Bystander Intervention Intentions
    a. Instructions: If you witnessed a fellow coworker or student experiencing behavior that could be construed as sexual harassment, rate the likelihood that you would engage in each behavior below.
    b. Scale

| Not at all likely (1) | Not very likely (2) | Somewhat likely (3) | Likely (4) | Extremely likely (5) |
|---|---|---|---|---|

    c. Items
        i. Pretend that you did not witness the incident(s).
        ii. Report the incident to someone in a higher position.
        iii. Redirect the initiator (perpetrator) away from harassing behaviors/
        iv. Remove the target (victim) from the situation.
        v. Testify as a witness during an investigation of the matter.
        vi. Ask the initiator (perpetrator) to refrain from such behavior.
        vii. Listen to your fellow student's problem in dealing with the incident.

viii. Provide emotional support to the fellow employee/student.
ix. Show concern for the fellow employee/student regarding the incident(s).
x. Look out for the personal welfare of the fellow employee/student.

6. Demographics
   a. What is your gender?
      i. Man
      ii. Woman
      iii. Non-binary
      iv. Other
   b. What is your age?
   c. What is your highest level of education you have completed up to this date?
      i. High school diploma or equivalent
      ii. Associate's degree
      iii. Bachelor's degree
      iv. Master's Degree
      v. Ph.D., professional doctorate (e.g., JD, MD, DDS), or equivalent
      vi. Other___________
   d. Are you of Spanish, Hispanic, or Latinx origin?
      i. Yes
      ii. No
   e. Choose one or more races that you consider yourself to be (multiple options)
      i. White or Caucasian
      ii. Black or African American
      iii. American Indian/Native American or Alaska Native
      iv. Asian/Asian American
      v. Native Hawaiian or Pacific Islander
      vi. Middle Eastern/Norther African (MENA)
      vii. Prefer not to say
      viii. Other
   f. Are you
      i. Employed for pay full time (30 hours or more per week)
      ii. Employed for pay part-time (less than 30 hours per week
      iii. A seasonal or "gig" worker
      iv. Not employed
      v. Other
7. Debriefing statement – Study 1

Thank you for participating in our research.  We are assessing the effectiveness of the abbreviated empathy training program on empathic concern and bystander intentions. We tested three different forms of training conditions for experiencing or recalling a sexual harassment story that you or someone you may know. The three different forms of training conditions included sexual harassment empathy training, burglary empathy training, and time management training. Each of the three conditions that were tested in this study plays a crucial role in developing or furthering our research, and we personally thank you for taking the time to complete this survey. You may have experienced mild to intense emotions while taking part in this research, or it may have triggered memories of similar experiences you may have had.  If you would like to speak to a confidential source, go to RAINN.org.
